# Decision-centered design of a clinical decision support system for acute management of pediatric congenital heart disease

**DOI:** 10.3389/fdgth.2022.1016522

**Published:** 2022-11-14

**Authors:** Azadeh Assadi, Peter C. Laussen, Gabrielle Freire, Marzyeh Ghassemi, Patricia Trbovich

**Affiliations:** ^1^ Department of Critical Care Medicine, Labatt Family Heart Centre, Toronto, ON, Canada; ^2^ HumanEra, Institute of Biomaterials and Biomedical Engineering, Department of Engineering and Applied Sciences, University of Toronto, Toronto, ON, Canada; ^3^ Institute of Medical Sciences, University of Toronto, Toronto, ON, Canada; ^4^ Executive Vice President for Health Affairs, Boston Children’s Hospital, Boston, MA, United States; ^5^ Department of Anesthesia, Anaesthesia, Harvard Medical School, Boston, MA, United States; ^6^ Division of Emergency Medicine, Department of Pediatrics, University of Toronto, Toronto, ON, Canada; ^7^ Electrical Engineering and Computer Science (EECS), Massachusetts Institute of Technology, Boston, MA, United States; ^8^ Institute for Medical Engineering & Science (IMES), Massachusetts Institute of Technology, Boston, MA, United States; ^9^ Vector Institute, Toronto, ON, Canada; ^10^ Canadian CIFAR AI Chair, Canada; ^11^ Institute of Health Policy Management and Evaluation, University of Toronto, Toronto, ON, Canada; ^12^ Research and Innovation, North York General Hospital, Toronto, ON, Canada

**Keywords:** decision support, congenital heart disease, macrocognition, emergency medicine, cognitive task analysis, digital health, decision centered design

## Abstract

**Background and Objectives:**

Children with congenital heart disease (CHD), have fragile hemodynamics and can deteriorate due to common childhood illnesses and the natural progression of their disease. During these acute periods of deterioration, these children often present to their local emergency departments (ED) where expertise in CHD is limited, and appropriate intervention is crucial to their survival. Previous studies identified that determining the appropriate intervention for CHD patients can be difficult for ED physicians, particularly since key components of effective decision making are not being met. Although key components of effective decision making for ED physicians have been identified, they have yet to be transformed into actionable guidance. We used decision centered design (DCD) to translate key components of decision making into decision requirements and associated design concepts, that we subsequently incorporated into a prototype clinical decision support system (CDSS).

**Methods:**

Using framework analysis, transcripts from Critical Decision Method interviews of CHD experts and ED physicians were inductively coded to identify key decision requirements for ED physicians that are currently not well supported, and their associated design concepts. A design workshop was held to refine the identified key decision requirements and design concepts as well as to sketch information that would satisfy the identified requirements. These were iteratively incorporated into a prototype CDSS.

**Results:**

Three decision requirements: (1) *distinguish the patient's unique physiology based on their unique cardiac anatomy*, (2) *explicitly consider CHD specific differential diagnoses to allow a more structured reflection of diagnosis*, and (3) *select CHD appropriate interventions for each patient*, were identified. These requirements along with design concepts and information needs identified through the design workshop were incorporated into the CDSS prototype.

**Conclusion:**

We identified key decision requirements and associated design concepts, that informed the design of a CDSS to provide actionable guidance for ED physicians when managing CHD patients. Meeting ED physicians' decision components with a CDSS requires the translation of their key decision requirements in its design. If not, we risk creating designs that interfere with clinician performance.

## 1. Introduction

Congenital heart disease (CHD) refers to the constellation of structural and functional defects of the heart that children are born with. There are a wide variety of anatomic differences in CHD, each with a different effect on blood flow through the body, heart, and lungs. These variations in blood flow and heart function are often referred to as the cardiac physiology of patients and can alter in response to common childhood illnesses and therapeutic interventions. The unique cardiac physiologies of these patients are the underlying cause of their cardiorespiratory deterioration and acute presentation to local emergency departments (ED). Timely and appropriate understanding of these physiologies and initiation of appropriate intervention is central to achieving optimal outcomes. The expertise required to correctly identify these physiologies and decide on a treatment strategy is often limited in the ED (1). As such, further CHD expertise is often sought to facilitate an understanding of the patient's physiology, expected baseline, and treatment strategies before transferring the patient to the cardiology service (2). In-person CHD expertise, however, is often lacking in community EDs which are often the initial site of care for acutely ill patients with CHD.

In this manuscript, we will build on our previous work and present the analysis and representation as well as the application design phases of the Decision Centered Design (DCD) framework to developing a clinical decision support system (CDSS)*.* DCD is a design framework that focuses on supporting decision making during time constrained, challenging, and uncertain situations where decision support is arguably most needed (3). DCD uses cognitive task analysis (CTA) [a set of methods to elicit, explain, and represent the mental processes involved in performing a task (4)] to identify key decisions and translate them into decision requirements that guide the intervention design (3). DCD was first used by a research program in the American navy called Tactical Decision Making Under Stress (TADMUS), to modify the design of displays to convey information clearly and facilitate decision making during time-pressured, high risk settings (3). In medicine, this framework has been used to design the following: a patient information visualization system to support chronic pain management (5), a decision support tool for airway management (6), colorectal cancer screening (7), and diagnostic support for family physicians (8). The DCD framework consists of five stages which are described in Table 1. Specifically in this manuscript, we focus on transforming the un-met decision components of ED physicians (described below) into key decision requirements and associated design concepts that will be used to produce a prototype CDSS called MyHeartPass™. MyHeartPass™'s design will support treatment related decision making among ED physicians managing acutely ill pediatric patients with CHD.

**Table 1:** Description of stages in decision centered design (3).

Stage	Stage description
Preparation	Become familiar and gather background information about the domain, distinguish characteristics of user groups, and begin to **identify cognitively complex elements of the task**
Knowledge elicitation	Use cognitive task analysis methods to elicit critical incidents and **key components** (e.g., goals, assessments, critical cues, and strategies) **of expert decision making**
Analysis and representation	Translate key components into **key decision requirements** to highlight key elements that will drive design. A decision requirements table is generated at this stage
Application design	Sketch **design concepts** using the decision requirements identified in the previous stage. Prototypes to support decision making are developed during this phase
Evaluation	**Assess whether the design supports decision making** in the context of the originally identified challenging scenarios using scenario-based approaches

## 2. Previous work

In a previous study, we identified key components of decision making for ED physicians that were not being met (2). Specifically, we used Critical Decision Method (CDM) to identify differences between ED physicians and subject matter experts (CHD experts) in how they acquire data and construct mental models to interpret patient data and determine course of therapy (2). CDM is a CTA technique used to study challenging incidents by eliciting concreate assessment indicators (e.g., cues and patterns) to treat incidents, particularly those that might have been missed by less experienced personnel (9). This method requires participants to retrospectively recount events from their perspective to elicit knowledge from working in challenging and atypical complex situations (10, 11). To understand the decision-making differences between CHD experts and ED physicians, clinicians from each specialty participated in a virtual, audio recorded, semi-structured, two-hour interview where they recalled details of a prior clinical encounter managing an acutely ill pediatric patient with CHD. Details of the study can be found in (2) and sample questions for the semi-structured interview can be found in (Supplementary Material S1). Transcripts of these interviews were used to compare the macrocognitive processes of CHD experts and ED physicians during their encounter. Macrocognition refers to the study of mental functions involved in acquisition, storage, interpretation, manipulation of knowledge used to perform a task under conditions of risk, time pressure, and uncertainty (12, 13). When managing CHD patients, we found the following components of ED physicians' effective decision making were not met: (i) Sensemaking—Visualizing structural heart defects to inform understanding of physiology, (ii) Anticipation—Identifying the CHD specific causes of deterioration, and (iii) Managing Complexity—Knowing the appropriate therapeutic intervention (2). Determining the appropriate therapeutic intervention for CHD patients was the most common concern of ED physicians and they always sought consultation in this regard from CHD experts (2). These key components of effective decision making have yet to be transformed into actionable guidance in support of ED physicians' management of CHD patients. A CDSS can be used to support ED physicians' CHD specific, treatment related, decision making. In this study, we use DCD to develop a CDSS to support ED physicians with key clinical decision requirements when managing patients with CHD.

## 3. Materials and methods

Institutional Research Ethics Board approval was obtained for this study prior to recruitment (REB#1000064567). Methodological details for the previously published *preparation* and *knowledge elicitation* phase have been described in (2). This study is part of a larger protocol previously published in (14).

### 3.1. Participants and setting

Participants were recruited from a large pediatric academic center with a university affiliated research and training program. This hospital is also a major cardiac specialty center with an annual average of 520 pediatric cardiac surgeries and a Pediatric Cardiac Intensive Care Unit (PCICU) that cares for these patients at various stages of their critical illness. The emergency department of this hospital sees over 50,000 pediatric patients and has 6,000 admissions each year (15). Pediatric cardiac intensivists from this hospital's PCICU and ED staff physicians for the emergency department were recruited as the CHD experts (EXP##) and ED physicians (PEM##) for this study. Rolling recruitment was email based and continued until saturation was reached and no new themes emerged thereafter.

### 3.2. Analysis and representation phase

A modified framework analysis was used to identify key decision requirements from the CDM interview transcripts described above*.* Framework analysis is a form of thematic analysis that uses inductively or deductively derived themes from data descriptions and abstractions (16, 17). This is achieved through 4 steps: data familiarization, framework identification, indexing, and charting.

• Data familiarization: To become familiar with the data, transcripts of the previously identified key decision components were reviewed by AA and TR who had extensive experience coding interview data.
• Framework identification: To create the analysis framework, each coder independently analyzed the data and inductively generated codes that identified key decision requirements and design concepts. The kinds of data, knowledge, and experience used by clinicians, as well as the purpose of their use in deciding therapeutic interventions were some of the features that were coded for. Coders then reviewed their codes to identify overlaps and variations. Where there were differences in the choice of codes describing similar features, coders modified their coding system to achieve consistency with each other. The analytical framework was then developed with the agreed upon codes.
• Indexing: The analytical framework was applied independently by both coders to transcripts one at a time allowing for coders to iteratively reviewed their codes for discrepancies until reaching consensus and a Cohen's Kappa of 0.72. At this point, the remainder of the transcripts were coded by a single coder (AA).
• Charting: Findings were summarized into a framework analysis matrix with unifying decision requirements and design concepts. To ensure the clinical accuracy of the findings, the identified key decision requirements and design concepts were reviewed by a CHD expert (OZ) external to the study team.

To create the decision requirements table, the previously identified decision components, as well as the newly identified key decision requirements and design concepts were consolidated. This decision requirements table was then used to design the prototype CDSS in the next phase.

### 3.3. Prototype design phase

To refine the key decision requirements and design concepts identified in the *Analysis and Representation phase,* a half-day design workshop was held with 5 CHD experts and 2 ED physicians. As participants in the design workshop did not have any previous experience in sketching prototypes, a brief orientation to the procedures, goals, and desired outcomes of the session was held at the outset. Participants were also provided a hypothetical clinical scenario to use as their reference during the workshop. As they worked through the hypothetical case, all members were guided to work collaboratively and sketch a list of information needs that would support the identified key decision requirements and design concepts. Group consensus was achieved on the first iteration of the prototype. Three subsequent iterations were completed to determine optimal layout of the User Interface (UI) which were each reviewed with a senior, well experienced CHD expert (PL) to ensure accuracy and relevance of content.

The CDSS prototype was iteratively designed by a researcher with human factors and medical training who was immersed in all phases of the DCD, and therefore had a solid understanding of the requirements. The first iterations were developed using Microsoft Excel®. Once the mockup UI was finalized, the CDSS was developed using Microsoft Visual Studio® using C#. The database for this prototype was designed using Microsoft SQL Server®.

## 4. Results

In total, 13 transcripts (6 CHD experts and 7 ED physicians) were analyzed using Framework Analysis. A summary of these findings is presented in Table 2 and described in greater detail in the subsections that follow.

**Table 2:** Decision requirements and design concepts table to inform the design of *MyHeartPass™* prototype.

Decision component	Decision requirement	Design concepts
(i) Sensemaking— Visualizing structural heart defects to inform understanding of physiology	• Distinguish the patient's unique physiology based on their cardiac anatomy “*I think about what the actual anatomy is and the resulting physiology. So, I think about the anatomic drivers for the current state of the patient from a cardiac standpoint.” (EXP03)* “*if you get a little bit of lead time in emerge, you get a call from cardio saying, hey, we've got a sick X coming in. And I say, great, can you remind me their physiology” (PEM06)*	Present a visual graphic of what the patient's heart looks like at current state “*I want some type of map that's like, you know, return to the heart, like how that connects to pulmonary circulation, how pulmonary circulation connects to heart, how heart connects to body. And if I can understand that path, that's helpful” (PEM02)* Include a description of the anatomy and the surgical interventions “*And then for many of us, even just decoding exactly what their anatomy is and like making that mental model of exactly what's going on” (PEM02)* Include the latest echocardiography report “*then I'm looking at their most recent echo results, if I have any, like, did this kid have really bad function like 10 percent ejection?” (PEM06)* Make patient specific baseline vital signs and a sample tracing of the patient's baseline ECG in a single view on a CDSS “*I'm looking for what are the baseline vital signs? So is this kid normally tachycardic or are they normally what's their normal oxygen sat? What's the normal BP? What's in our normal vital signs?” (PEM06)* Include a description of possible physiological states based on patient's unique CHD anatomy Include a description of possible signs and symptoms as well as investigations to ascertain the relevance of a physiology “*I like these lists, kind of envision how that child would present” (PEM03)*
(ii) *Anticipation*—Identifying the CHD specific causes of deterioration	• Explicitly consider CHD specific differential diagnoses to allow a more structured reflection of diagnosis “*you have this map of their heart disease and the surgery they had, their residual lesion or function … and the next step is thinking of what the problem could be… like, AVSD I think AV valve might be a problem” (EXP01)*	7. Include a list of possible CHD specific diagnoses and how they can be evaluated “*What could happen based on all the data I'm seeing, based on a population of patients and based on that individual patient and the Information that was available at the time” (EXP01)*
(iii) *Managing Complexity*—Identifying the appropriate therapeutic intervention	• Select CHD appropriate interventions for each patient “*for the management, I really kind of do oftentimes think about the physiology that I manage at the bedside in terms of just like a bucket of stuff that we need to do for a specific physiology.” (EXP03)*	8. Include a list of physiology based therapeutic recommendations and considerations “*In the moment, you just go through the algorithm… for the (spelling) Tets, need some opioids, some beta blockers, fluids, that sort of thing” (PEM06)* 9. Include contact information for clinicians to use for further assistance from CHD experts

CDSS, Clinical Decision Support System; CHD, Congenital Heart Disease; ECG, electrocardiogram.

This study focuses on the *analysis and representation* as well as the *application design* phase of the DCD framework. As such, in section 3.1 *Analysis and Representation*, we will present a detailed description of each decision requirement and its associated design concepts based on the analysis of our interview transcripts. In section 3.2 *Application design,* we will describe the application that was designed and developed based on our findings during *Analysis and Representation* (Table 2) and the design workshop.

### 4.1. Analysis and representation

#### 4.1.1. Decision requirements

The analysis of our interview transcripts resulted in identification of decision requirements aligned with each of the three previously identified un-met key decision components: (i) Sensemaking—Visualizing structural heart defects to inform understanding of physiology, (ii) Anticipation—Identifying the CHD specific causes of deterioration, and (iii) Managing Complexity—Knowing the appropriate therapeutic intervention (2). The details of each decision requirement are described below.

##### 4.1.1.1. Distinguish the patient's unique physiology based on their unique cardiac anatomy

To support the decision component of visualization of cardiac anatomy and (Sensemaking), we identified the following decision requirement for ED physicians: *distinguish the patient's unique physiology based on their cardiac anatomy*.

There is a wide range of anatomic variations among patients with different CHDs. The baseline cardiac physiology of patients varies with their unique CHD anatomy, patterns of blood flow through the heart and lungs, as well as the quality of their heart's relaxation and squeeze. This baseline cardiac physiology is often altered in response to intercurrent illness, changes in the anatomy or function of the heart, its valves, muscle, and great vessels, as well as the lungs and their function. Clinicians' understanding of patient's unique cardiac anatomy and its impact on cardiac physiology is crucial in appropriately managing these patients. For example, EXP02 (a CHD expert) and PEM07 (an ED physician) both described cases that reflected a physiology of poor pulmonary blood flow. This physiology can occur in children who have a higher resistance in their lungs which limit the amount of blood that goes through them at normal pressures (example given by EXP02) or when there is an anatomic obstruction to blood leaving the right heart and going through the great vessel connecting the right heart to the lungs (example given by PEM07). An understanding of patients' cardiac anatomy and physiology in each of these cases is central to the appropriate management of these patients. In the example of the patient described by EXP02, the knowledge of greater resistance in the lungs and the resulting poor blood flow through them allowed for treatment interventions to be targeted towards decreasing the resistance in the lungs. In the example provided by PEM07 on the other hand, without the understanding of the anatomic obstruction to pulmonary blood flow, treatments primarily targeted a lung pathology instead of optimizing blood flow past the anatomic obstruction. Understanding the cardiac anatomy and physiology also helps with understanding acceptable vital signs and patient's risk and mechanism for further deterioration and cardiorespiratory collapse which would dictate the type and urgency of treatment choices.

CHD experts described using an understanding of the patient's unique CHD anatomy to ascertain appropriate vital sign goals, possible diagnoses, cardiac physiologies, and interventions. Using the cardiac physiology of patients was the hallmark of CHD experts' sensemaking and decision making. To conceptualize CHD patients' anatomy and physiology, CHD experts used patients' clinical data (e.g., patient anatomy, vital signs, physical exam, presenting history, response to treatment, previous cardiac evaluations, etc.), their expertise in cardiac imaging, their knowledge of the natural history of various CHD, as well as their experience managing a variety of acutely ill pediatric CHD patients. However, visualizing a patient's CHD was difficult for ED physicians who did not encounter these patients as frequently as CHD experts. The heavy use of acronyms and non-descriptive procedural names in echocardiography reports and clinical notes are some of the reasons for which visualization of the cardiac anatomy may be impaired among ED physicians. ED physicians did not consistently conceptualize and analyze patients' cardiac physiology to determine treatment. ED physicians expressed difficulties conceptualizing unique patient CHDs and lacked the experience or knowledge base to readily interpret clinical data to determine patients' physiologies and appropriate treatment. They also described spending time looking through the patient EHR to find patient's unique acceptable baseline vital signs and/or referring to parents for this information, which can be limited by parental language and knowledge barriers.

**Design Concepts:**

1. Present a visual graphic of what the patient's heart looks like at current state: CHD experts described creating a visual image of a patient's cardiac anatomy which they used to ascertain their cardiac physiology. These visual images were not always anatomically precise but were accurate in depicting blood flow and the function of the heart and its great vessels. ED physicians, however, had a harder time conceptualizing the anatomy and associated physiology. They described referring to alternative sources (e.g., Google) to obtain a “generic” image of the cardiac anatomy of their patient.
2. Include a description of the anatomy and the surgical interventions: Both CHD experts and ED physicians used the written description of the patient's cardiac anatomy and surgical interventions that were found in their EHR as the reference based on which they visualized patient's anatomy.
3. Include the latest echocardiography report: CHD experts used patients' latest echocardiography to ascertain the function of the heart muscle and its various valves which they used in conceptualizing the patient's unique CHD and determining their physiology. ED physicians also referred to this report but generally only used the findings on the function of the heart muscle in how they conceptualized the patient and made treatment decisions.
4. Make patient specific baseline vital signs and a sample tracing of the patient's baseline ECG in a single view on a CDSS: CHD experts used their experience and knowledge in CHD to ascertain patient's acceptable baseline vital signs but occasionally did refer to the patient's EHR to review patient's baseline ECG. ED physicians, however, relied on the patient EHR to find acceptable baseline vital signs and ECG. This process was described to be very time consuming and difficult.
5. Include a description of possible physiological states based on patient's unique CHD anatomy: CHD experts relied on their experience to determine patient's physiology based on an understanding of their anatomy. ED physicians, however, had a difficult time determining the cardiac physiology of these patients based on anatomy alone.
6. Include a description of possible signs and symptoms as well as investigations to ascertain the relevance of a physiology: CHD experts were able to use their experience to quickly evaluate the relevance of a physiology in a patient and conducted various investigations to identify the most suitable physiology for a patient in a given state. ED physicians relied on CHD experts for this determination.

##### 4.1.1.2. Explicitly consider CHD specific differential diagnoses to allow a more structured reflection of diagnosis

To support the decision component of identifying CHD specific causes of deterioration in CHD patients (Anticipation), we identified the following decision requirement for ED physicians: *explicitly consider CHD specific differential diagnoses to allow a more structured reflection of diagnosis*.

Differential diagnoses structure the types and priorities in which diagnostics and interventions are initiated. Considering CHD specific differential diagnoses early in the patient-clinician encounter is crucial in providing early and appropriate treatment as well as appreciating risk for further deterioration. For example, EXP01 and PEM05 physician recalled patients presenting with signs of severe infection and fast breathing. In these cases, including an increase in leakiness of a heart valve (specifically the left atrioventricular valve) following surgeries that involve these valves (such as atrioventricular septal defect repair) as part of the differential to explain the patient's fast breathing is crucial. This consideration by EXP01, ensured that this valve was imaged to ensure its integrity and triggered the clinician to support the heart and minimize any potential worsening leakiness while treating the infectious trigger to presentation. These measures included early use of medications to support the function of the heart and decrease systemic resistance to facilitate better cardiac output. It also included the use of sedatives to decrease oxygen consumption and ventilation strategies to improve cardiac output while also decreasing the associated work of breathing. In the absence of this CHD specific consideration, the therapeutic measures taken by PEM05 were focused on supporting the infectious prodrome and the patient hemodynamics. These measures included the careful use of fluids and ventilation strategies to support the work of breathing and optimize oxygenation. Suspecting the correct CHD specific differential is important as treatment of heart failure would vary depending on the specific cardiac differential (e.g., treating left heart failure is different from right heart failure). CHD experts were readily able to include CHD specific differentials on their list of possible diagnoses based on their clinical examination and basic investigations before obtaining definitive cardiac imaging. They relied on their ability to conceptualize cardiac physiology, clinical exam, patient history, and their knowledge and experience of the natural history of specific CHDs to suspect appropriate differentials. While ED physicians always suspected heart failure, they had difficulties generating CHD specific differentials. ED physicians reported that the reasons they struggled to generate CHD specific differentials included: difficulties conceptualizing CHD specific physiologies, limited knowledge of the natural history of each CHD and common residual lesions, and lack of familiarity with presentation patterns of CHD specific differentials.

**Design Concepts:**

7. Include a list of possible CHD specific diagnoses and how they can be evaluated: CHD experts had an intuitive list of potential CHD specific differential diagnoses and how to evaluate their relevance to each patient. This list they had developed through experience and was not known to ED physicians.

##### 4.1.1.3. Select CHD appropriate interventions for each patient

To support the decision component of identifying appropriate therapeutic interventions in CHD patients (Managing Complexity), we identified the following decision requirement for ED physicians: *select CHD appropriate interventions for each patient*.

Patients with CHD have fragile hemodynamics and can deteriorate rapidly if untreated or improperly treated at the time of their acute presentation. This is particularly important as some of the standard treatment strategies for acutely ill children without CHD can lead to further deterioration and cardiorespiratory collapse in children with certain CHDs. For example, EXP04 described a patient whose heart did not squeeze very well. For this patient, EXP04 explained that while administration of fluids to improve blood pressure is common and effective in children without CHD, it could worsen heart failure in children with certain CHDs such as those whose heart did not squeeze very well. Conversely, certain other CHDs (e.g., patients with Fontan circulation) would benefit from fluid administration as their circulation suffers significantly with dehydration or low intravascular fluid status. For example, the patient described by PEM03, had a Fontan circulation, and benefitted from volume administration at the recommendation of consulting CHD experts. Therefore, recognizing the appropriate therapy for each patient with CHD is crucial in the survival and recovery of these patients. CHD experts drew from their understanding of the patient's unique physiology, as well as their experience and knowledge in treating these patients to determine the appropriate therapy. They also used the patient's response to therapy to further their understanding of the patient's physiology. ED physicians recognized the fragility of these patients both in the face of illness and in response to treatment and were often unsure of the most appropriate course of treatment. They based their treatment choices on ways to restore the patient's previous baseline physiological parameters and mostly relied on CHD experts to direct the care of these patients. This could delay delivery of appropriate treatment either due to delays in seeking CHD expertise or delays in response from CHD expertise once consulted. Given the fragile hemodynamics of these patients, the consequences of delays can lead to further deterioration and cardiovascular collapse.

**Design Concepts:**

8. Include a list of physiology based therapeutic recommendations and considerations: CHD physicians made physiology based therapeutic recommendations which they knew based on their training and expertise. This was not always known to ED physicians which made deciding interventions more challenging and understanding the CHD recommended interventions difficult.
9. Include contact information for clinicians to use for further assistance from CHD experts: ED physicians appropriately always sought additional CHD expertise when encountering CHD patients. The incorporation of this contact information would facilitate this process for ED physicians from community EDs while also serving as a reminder to consider expert consultation on these children.

### 4.2. Application design

**Figure 1:**
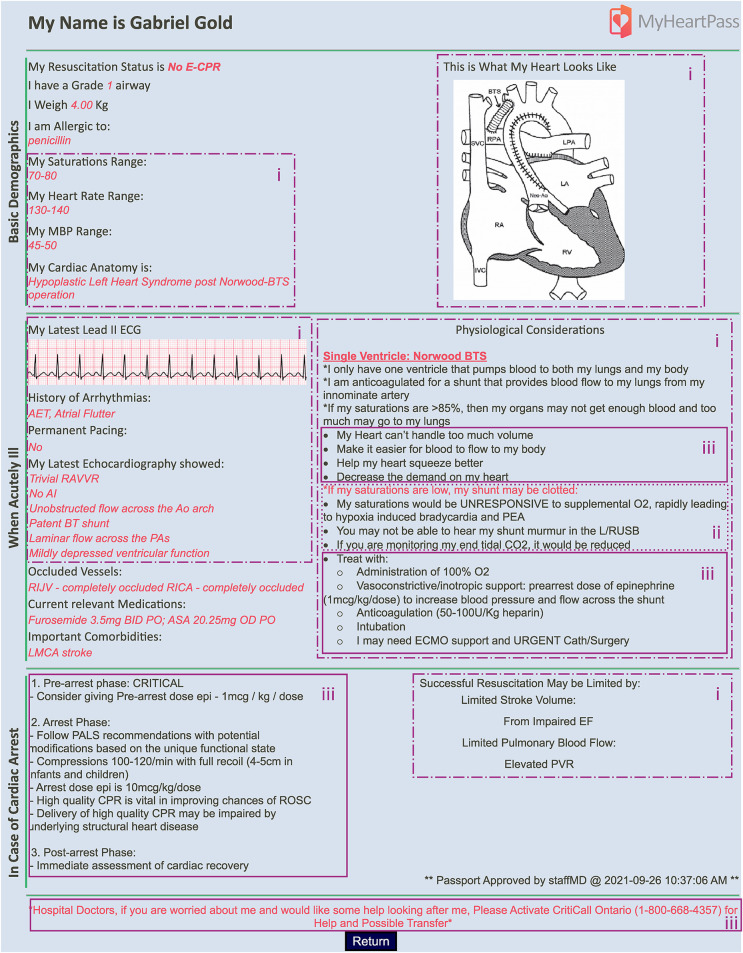
Screenshot of *MyHeartPass™* prototype. Sections marked with (i) refer to elements of design pertaining to key decision requirement *distinguish the patient's unique physiology based on their unique cardiac anatomy.* Elements marked with (ii) capture the elements pertaining to the key decision requirement of *explicitly consider CHD specific differential diagnoses to allow a more structured reflection of diagnosis;* and (iii) capture the elements pertaining to the key decision requirement of *select CHD appropriate interventions for each patient*.

The prototype CDSS, called *MyHeartPass™* (Figure 1), was designed based on the decision requirements and design concepts table (Table 2) described in the previous section and the design workshop. *MyHeartPass™* was designed to support the three key decision requirements identified in the *Analysis and Representation* phase. To support ED physicians, *distinguish the patient's unique physiology based on their unique cardiac anatomy*, an anatomic diagram of the patient's current heart (Design Concept 1 in Table 2) and a description of their cardiac anatomy (Design Concept 2 in Table 2) were included in the CDSS. The patient's latest echocardiographic report (Design Concept 3 in Table 2) was also included to facilitate an understanding of the function of the heart and its structures. Patient's unique acceptable range of vital signs and ECG tracing (Design Concept 4 in Table 2) were also included. A description of patient's unique baseline physiology and its features (Design Concept 5 and 6 in Table 2) were also incorporated into the CDSS. These design concepts are highlighted in Figure 1 with dashed boxes marked with “*i*”. To facilitate *explicit consideration of CHD specific differential diagnoses to allow a more structured reflection of diagnosis*, some high-risk CHD specific differentials were included in the CDSS along with diagnostic considerations to determine the relevance of this diagnosis to the patient (Design Concept 7 in Table 2). These design concepts are identified with *“ii*” in Figure 1. Finally, to *select CHD appropriate interventions for each patient* in the ED, a list of physiology based therapeutic considerations (Design Concept 8 in Table 2) and instructions on how CHD expertise can be reached within the province (Design Concept 9 in Table 2) were incorporated into the CDSS. Design concepts pertaining to this key decision requirement are identified in Figure 1 with “*iii*”. In addition to content from Table 2, information that would identify the patient, such as their name and basic demographics was also incorporated in the prototype along with information on current medications, and obstructed vessels. These were the result of the design workshop to improve the overall design of the CDSS.

## 5. Discussions

In this study, we applied a DCD framework to design a CDSS to support clinical decision making in the ED. In a survey of all ED physicians in the state of Michigan, Cashen and colleagues found that nearly 58% of these physicians were worried and uncomfortable caring for patients with single ventricle physiology (1). When asked about the expected saturations for these patients, 52% of general ED physicians and 35% of pediatric ED physicians were unsure of their response while 18% of general ED physicians and 26% of pediatric ED physicians expressed an incorrect saturation expectation (1). We built on our previous work that identified that key decision components in ED physicians' decision making for managing acutely ill pediatric CHD patients are not adequately met. To date, no study has characterized decision requirements needed to fill these unmet needs. Filling this gap, we applied a DCD framework to identify the key decision requirements and associated design concepts to iteratively develop our prototype CDSS called *MyHeartPass™.* Specifically, the 3 key decision requirements that we identified were: (i) distinguish the patient's unique physiology based on their unique cardiac anatomy, (ii) explicitly consider CHD specific differential diagnoses to allow a more structured reflection of diagnosis, and (iii) select CHD appropriate interventions for each patient. Conceptualizing patients' unique cardiac anatomy allows clinicians to understand the baseline cardiac physiology of these patients which informs their understanding of expected baseline vital signs and hemodynamic fragility as well as an expectation of how they could respond to various interventions. This understanding of patient anatomy and physiology also facilitates an understanding of potential cardiac related differential diagnoses which also influence the choice of therapeutic interventions. In addition to this understanding, the ability to identify the appropriate treatment relies on an inherent knowledge of available interventions and how those interventions are tolerated in children with different CHDs. We also found that the hallmark of decision making for CHD experts was physiology-based diagnosis and treatment. A feature that was underutilized in ED physicians' decision making when treating patients with CHD.

Expertise in pediatric CHD is limited in the ED (1), particularly in community EDs. This is due to multiple reasons including variable mandatory CHD training for ED physicians (18, 19), limited experience managing these patients due to their relative infrequent encounter compared to other pediatric conditions (1, 20), and limited availability of in-person CHD expertise in community EDs. Nevertheless, the population of patients with CHD who are living in diverse communities and away from specialty heart centers is growing and their presentations to ED are projected to increase (1, 20, 21). The complexity and hemodynamic fragility of these patients places them at higher risk for severe deterioration in the face of common intercurrent childhood illnesses (20, 22–24). Therefore, to prevent deterioration and cardiorespiratory arrest, appropriate CHD specific treatments must be initiated in parallel to treating the intercurrent illness (22–24). Clinicians' *sensemaking,* particularly their ability to conceptualize the cardiac anatomy and its subsequent physiology is central to not only understanding a CHD patient's current state, but also *anticipating* their cause and mechanism of deterioration, and *managing complexity* in treating these patients. Supporting ED physicians in these key decision components is therefore crucial in optimizing appropriate treatment and preventing further deterioration. *MyHeartPass™,* the prototype we developed in this study, offers a promising approach for providing ED physicians with the information they need to make appropriate treatment related decisions for acutely ill pediatric patients with CHD. Its DCD provides clinicians with focused information that aims to improve clinicians' sensemaking and anticipation as they manage the complexity in providing these patients with the most appropriate therapeutic interventions.

Compared with the commonly used user-centred design framework which focuses on designing for specific tasks performed by the end user, the DCD framework focuses on identifying and developing decision supports that can assist users with challenging tasks. DCD has previously been applied in the design of medical CDSS (5–8). Our use of the DCD framework to identify key decision components of ED physicians based on a comparison with the cognitive processes of CHD experts, to our knowledge, has not been previously reported. The DCD framework draws attention to the significance of the misalignment between current information ED physicians have access to and the information they require to manage CHD patients. This design could facilitate consultations with CHD experts by providing information that was deemed pertinent by both clinicians on the same platform.

The main focus of this paper was to develop a CDSS to support ED physicians' management of CHD patients based on decision requirements. The detailed results of the assessment of the effectiveness of the CDSS in addressing unmet ED physician needs will be reported separately. The DCD framework provides a critical first step in identifying ED physician decision requirements and applying them to the design of a CDSS. We anticipate that the DCD framework will be helpful for similar studies focused on developing clinical CDSS where close collaborative relationships between clinicians of different subspecialties is required to best manage patients.

At the time of writing this manuscript, a case-based simulation study was underway to evaluate the effect of *MyHeartPass™* on treatment related decision making among ED physicians. Future work will need to look at how *MyHeartPass™* can be integrated into the existing EHR such that it is automatically updated with relevant EHR data (intended design concept) and how it could be accessed either directly from the EHR or as a separate application that would best fit the workflow of each ED.

## 6. Limitations

This study was conducted as a single center study which made the sample size small. As this was also a major cardiac center, the existing referral structures and greater CHD exposure of ED physicians may have influenced the findings of this study. The study also focused on pediatric ED physicians who are not only more experienced in caring for acutely ill pediatric patients, but also have had more exposure to pediatric patients with CHD during their training. Further studies of general ED physicians' decision making are required to identify any differences in their key decision components that may affect the DCD of *MyHeartPass™.* There may have also been some recall bias associated to our CTA study as participants were asked to recall a scenario from memory. We don't anticipate this had a significant impact on the design of *MyHeartPass™* as CDM focuses on key decisions instead of precise details.

## 7. Conclusion

The objective for this study was to apply a DCD framework to the development of a prototype CDSS that supports ED physicians in deciding on the appropriate therapeutic intervention for pediatric patients with CHD who present to the ED with acute illness. Through this design framework, we identified the following 3 previously unsupported key decision requirements: (i) distinguish the patient's unique physiology based on their unique cardiac anatomy, (ii) explicitly consider CHD specific differential diagnoses to allow a more structured reflection of diagnosis, and (iii) select CHD appropriate interventions for each patient. This rigorous design process ensured the translation of key decision requirements into design concepts of the CDSS to provide actionable guidance for ED physicians when managing CHD patients.

## Data Availability

The datasets presented in this article are not readily available because the study's REB mandates the transcript data to remain behind institutional firewall at all times. Requests to access the datasets should be directed to az.assadi@mail.utoronto.ca.

## References

[1] Cashen K Gupta P Lieh-Lai M Mastropietro C. Infants with single ventricle physiology in the emergency department: are physicians prepared? J Pediatr. (2011) 159(2):273–7.e1. 10.1016/j.jpeds.2011.01.031 21392789

[2] Assadi A Laussen PC Freire G Trbovich P. Understanding clinician macrocognition to inform the design of a congenital heart disease clinical decision support system. Front Cardiovasc Med. (2022) 9:1–10. 10.3389/fcvm.2022.767378 PMC8850471 35187118

[3] Militello LG Klein G. Decision-Centered Design. In Lee JD, Kirlik A, editors. The Oxford Handbook of Cognitive Engineering, Oxford Library of Psychology. online edn, Oxford Academic (2003). 10.1093/oxfordhb/9780199757183.013.0016.

[4] Klein G Militello L. Some guidelines for conducting a cognitive task analysis. Adv Hum Perform Cogn Eng Res. (2001) 1:163–99. 10.1016/S1479-3601(01)01006-2

[5] Harle CA Diiulio J Downs SM Danielson EC Anders S Cook RL Decision-centered design of patient information visualizations to support chronic pain care. Appl Clin Inform. (2019) 1(4):719–28. 10.1055/s-0039-1696668 PMC6760988 31556075

[6] Schnittker R Marshall SD Horberry T Young K. Decision-centred design in healthcare: the process of identifying a decision support tool for airway management. Appl Ergon. (2019) 77:70–82. 10.1016/j.apergo.2019.01.005 30832780

[7] Militello LG Saleem JJ Borders MR Sushereba CE Haverkamp D Wolf SP Designing colorectal cancer screening decision support: a cognitive engineering enterprise. J Cogn Eng Decis Mak. (2016) 10(1):74–90. 10.1177/1555343416630875 26973441 PMC4784691

[8] Porat T Kostopoulou O Woolley A Delaney BC. Eliciting user decision requirements for designing computerized diagnostic support for family physicians. J Cogn Eng Decis Mak. (2016) 10(1):57–73. 10.1177/1555343415608973

[9] Hoffman RR Crandall B Shadbolt N. Use of the critical decision method to elicit expert knowledge: a case study in the methodology of cognitive task analysis. Hum Factors J Hum Factors Ergon Soc. (2007) 40(2):254–76. 10.1518/001872098779480442

[10] Crandall B Klein GA Hoffman RR. Working minds: A practitioner’s guide to cognitive task analysis. 1st ed. Cambridge: A Bradford Book (2006).

[11] Crandall B Getchell-Reiter K. Critical decision method: a technique for eliciting concrete assessment indicators from the intuition of NICU nurses. Adv Nurs Sci. (1993) 16(1):42–51. 10.1097/00012272-199309000-00006 8311424

[12] Klein G Wright C. Macrocognition: from theory to toolbox. Front Psychol. (2016) 7:1–5. 10.3389/fpsyg.2016.00054 26858684 PMC4731510

[13] Klein G Ross KG Moon BM Klein DE Hoffman RR Hollnagel E. Macrocognition. IEEE Intell Syst. (2003) 18(3):81–5. 10.1109/MIS.2003.1200735

[14] Assadi A Laussen P Trbovich P. Mixed-methods approach to understanding clinician macrocognition in the design of a clinical decision support tool: a study protocol. BMJ Open. (2020) 10(3):e035313. 10.1136/bmjopen-2019-035313 32213525 PMC7170622

[15] The Hospital for Sick Children. Paediatric emergency medicine. (2014) [cited 2018 Sep 24]. Available from: http://www.sickkids.ca/PaediatricEmergencyMedicine/index.html

[16] Goldsmith LJ. Using framework analysis in applied qualitative research. Qual Rep. (2021) 26(6):2061–76. 10.46743/2160-3715/2021.5011

[17] Gale NK Heath G Cameron E Rashid S Redwood S. Using the framework method for the analysis of qualitative data in multi-disciplinary health research. BMC Med Res Methodol. (2013) 7(5):260–1. 10.1186/1471-2288-13-117 PMC3848812 24047204

[18] Royal College of Physicians and Surgeons of Canada. Subspecialty training requirements in pediatric emergency medicin*e*. (2017) (1):2–3.

[19] Royal College of Physicians and Surgeons of Canada. Specialty training requirements in emergency medicine. (2014) (613):1–2.

[20] Edelson JB Rossano JW Griffis H Dai D Faerber J Ravishankar C Emergency department visits by children with congenital heart disease. J Am Coll Cardiol. (2018) 72(15):1817–25. 10.1016/j.jacc.2018.07.055 30286926

[21] Competence Network for Congenital Heart Defects. The national register for congenital heart defects. [cited 2021 Apr 12]. Available from: https://www.kompetenznetz-ahf.de/en/about-us/register/

[22] Lowry AW Knudson JD Cabrera AG Graves DE Morales DLS Rossano JW. Cardiopulmonary resuscitation in hospitalized children with cardiovascular disease: estimated prevalence and outcomes from the Kids’ Inpatient database. Pediatr Crit Care Med. (2013) 14(3):248–55. 10.1097/PCC.0b013e3182713329 23462352

[23] Peddy SB Hazinski MF Laussen PC Thiagarajan RR Hoffman GM Nadkarni V Cardiopulmonary resuscitation: special considerations for infants and children with cardiac disease. Cardiol Young. (2007) 17(Suppl. 2):116–26. 10.1017/S1047951107001229 18039405

[24] Marino BS Tabbutt S Maclaren G Hazinksi MF Adatia I Atkins DL Cardiopulmonary resuscitation in infants and children with cardiac disease: a scientific statement from the American Heart Association. Circulation. (2018) 137:e1–e92. 10.1161/CIR.0000000000000524 29685887

