# Supplementary material for: Decision-centered design of a clinical decision support system for acute management of pediatric congenital heart disease
Source: Front Digit Health. 2022 Nov 14;4:1016522. doi: 10.3389/fdgth.2022.1016522 (PMC9701701; doi:10.3389/fdgth.2022.1016522)
Supplement: Supplementary file 1 [file Table1.pdf]

Supplementary Material

**Table 1** - Cognitive Task Analysis Interview Guidelines		
**Phase**	**Description**	
	**Non-CHD expert**	**CHD expert**
Preamble	Thank participant for volunteering for the study and provide an overview of the structure of the interview, what specific areas are of interest, and how they will be guided through the interview. Introduction of study team/facilitators and their role<br>*e.g. Thank you for volunteering for this study that aims to understand how clinicians make decisions when it comes to managing pediatric CHD patients who present to them with acute illness. Specifically, I will ask you to reflect on how you formulate differential diagnoses and how you narrow them down to an initial diagnosis for a patient. I will also ask you to walk me through those thought processes and what kinds of information you rely on at each step. By understanding how clinicians make decisions, we hope to be able to design better clinical decision support systems that are more in tuned and aligned with the cognitive processes of their users. We will test this hypothesis on a CDSS prototype for CHD.*<br>*To start, we will go over some instructions, review some of the broad questions that this interview will try to ask and seek answers for, and sign consent forms before starting a recording of the interview. If you do not wish to be audio recorded, that's okay, we will be taking notes throughout our conversation. You will be guided through a discussion with some scenarios that will help establish a timeline and themes that would suggest a pattern of how you would approach these particular patients. The interview will last no more than 2 hours and we suspect it will be on average of 90 minutes long.*	
Introduction	Introduction to the study and description of how a semi-structured interview using CDM will operate. Consent for audio recording will be obtained and rules of anonymity reviewed before starting the interview.<br>*e.g. For this interview, a critical decision method will be used. This method seeks to gain an understanding of how someone made decisions, under conditions of uncertainty where the risk associated to decision was considered high. To get at this, you will be asked to recall a scenario from your past and describe the scenario in as much detail as possible without the use of patients' or colleagues' names who were involved in your scenario. You may use pseudo-names if you prefer. Once the interview is started, the discussion will be audio recorded and fields notes obtained to facilitate the discussion and future analysis. No names will be documented to ensure anonymity is maintained. Within hours of completing the interview, the audio file will be uploaded to a drive that is password protected and behind an organizational firewall. Do you have any questions or concerns before we start? .*	
Scenario Selection	*Please think about a clinical scenario from ED involving a child with CHD that was particularly challenging,*	*Please think about a clinical scenario involving a child with CHD from your practice as a cardiac intensivist that*

<table>
<tr><td></td><td>*and your clinical expertise and experience made a difference in how things turned out. For example, it could have been challenging because it was not clear that the history of CHD was relevant to the current presentation, or that the symptoms were not so much in keeping with CHD. It could be a case where you think if someone else had managed the patient instead of you, things would have turned out differently*</td><td>*was particularly challenging, and your clinical expertise and experience made a difference in how things turned out. For example, it could have been challenging because it was not clear that the history of CHD was relevant to the current presentation, or that the symptoms were not so much in keeping with CHD. It could be a case where you think if someone else had managed the patient instead of you, things would have turned out differently*</td></tr>
<tr><td>Event Recall</td><td colspan="2">Participants asked to describe the scenario in detail and in sequence where possible, to allow the creation of a timeline of events (particularly decision points)
e.g. Select questions from the list below</td></tr>
<tr><td rowspan="2">Creation of a Timeline and identification of decision points</td><td colspan="2">Interviewer will use the timeline of events they have created based on the participant narrative to verify the story and timeline. In particular, the location of decisions and kinds of decisions will be clearly identified for further probing</td></tr>
<tr><td>Special attention will be paid to whether/when the participant sought additional help/consultation from CHD experts available to them as well as whether they considered transferring the patient to a cardiac ICU or cardiology ward</td><td>Special attention will be paid to whether/when participants sought additional help from cardiovascular surgeons or other cardiologists, echocardiographers and interventionalists</td></tr>
<tr><td>Probing questions</td><td colspan="2">Interviewer will utilize a semi-structured interview format to ask probing questions (e.g. similar to Klein's questions posted below) to better understand the circumstances surrounding the decision points.
e.g. Additional questions about what patient specific data was needed to understand the physiology or to make decisions and decide on interventions; what specialized/domain specific knowledge was used/needed to make decisions and suggests treatments; was it possible to ask for further expertise and if so from where/whom; Why was that information/knowledge important; was it difficult to find this information; and if it effected their ability to make decisions and provide treatment</td></tr>
<tr><td>Closing</td><td colspan="2">Participants will be interviewed for a maximum of 105 min and allowed 15 min for any additional questions or comments. Once those questions/comments are addressed, they will be presented with contact information of the study team and provided a thank you gift card of $50 as a token of the team's appreciation for their time
e.g. There is just about 15 min left in the interview. If there are any final questions or comments, you are welcome to ask them. You can connect with the study team by this contact information at any time should any questions</td></tr>
</table>

	or concerns arise. Otherwise, with this gift card, please accept our gratitude for your time and input.

**Table 2 -** Modified Klien's Probing Questions		
**General Questions**		
	Data types, variables, etc.	
		What were you seeing?<br>What were you hearing?<br>If you were to explain what was going on with the patient to a novice, how would you do it/what would you say?<br>What was the physiology that you were seeing for this patient?<br>Why was the patient presenting like they did?
	Analogies to past cases	
		Were you reminded of previous similar experiences?<br>Which of them is most similar?
	Standard Scenarios	
		Is this case a typical scenario?<br>Is it the sort of scenario you were trained to deal with?<br>How many incidents like this have you been involved with?
**Decision point questions**		
	Available options	
		What other options were available?<br>What other options were considered?
	Rationale for selection of options	
		How was the option selected/rejected?<br>What rule were you following?<br>Were you conscious of making a decision?<br>What were you aiming to treat? What did you wish to achieve by your treatment?
	Shifts/changes in situational awareness	
		If you had to describe the situation to someone taking over on the next shift, how would you summarise it?<br>If you were to convey your concerns and thoughts to the nurse looking after the patient, what would you have said?
	Information requirements at given points	
		What information did you use in making this decision?<br>How was it obtained?<br>Did it take a long time to obtain this information? Why?<br>How did you know you needed that information?<br>What training or experience was necessary or helpful in making this decision?<br>How confident were you in the information?<br>Did you need additional help from others to obtain this information?<br>How critical was this information for you?
	Missing information	
		If the decision was not the optimal one, what knowledge or information that was not available might have helped?<br>Was timing to obtain information an issue/consideration?
	Speculation	

		If a particular feature had been different, how would it have affected your decision?<br>Would the patient's evolving state have affected your decision making or information seeking approach?
	Goals	
		What were your goals at this point?<br>What were your command objectives?
	Imagery/mental simulation	
		Did you imagine the consequences of the action?<br>Did you imagine the events that would transpire given the situation and information available?<br>Did you imagine your possible options and how they would each transpire if you pursued each of them?
	Errors	
		Did you acknowledge if your situation assessment or option selection were correct?<br>How might a novice have behaved differently?<br>What mistakes are likely at this point?<br>What else could this presentation resemble?
	Potential aiding	
		If decisions were not optimal, what knowledge, or information was missing which could have aided decision making?<br>If the rate of action seemed insufficient, what aids do you think could have helped in the process?<br>What additional expertise would you have liked to have had access to if any?<br>What additional diagnostics would you have liked?
	Decision making time/effort	
		How much time pressure was involved in making this decision?<br>How long did it actually take to make this decision?
